# Microbial Biofilms in the Food Industry—A Comprehensive Review

**DOI:** 10.3390/ijerph18042014

**Published:** 2021-02-19

**Authors:** Conrado Carrascosa, Dele Raheem, Fernando Ramos, Ariana Saraiva, António Raposo

**Affiliations:** 1 Department of Animal Pathology and Production, Bromatology and Food Technology, Faculty of Veterinary, Universidad de Las Palmas de Gran Canaria, Trasmontaña s/n, 35413 Arucas, Spain; ariana_23@outlook.pt; 2 Northern Institute for Environmental and Minority Law (NIEM), Arctic Centre, University of Lapland, 96101 Rovaniemi, Finland; braheem@ulapland.fi; 3 Pharmacy Faculty, University of Coimbra, Azinhaga de Santa Comba, 3000-548 Coimbra, Portugal; framos@ff.uc.pt; 4 REQUIMTE/LAQV, R. D. Manuel II, 55142 Apartado, Portugal; 5 CBIOS (Research Center for Biosciences and Health Technologies), Universidade Lusófona de Humanidades e Tecnologias, Campo Grande 376, 1749-024 Lisboa, Portugal

**Keywords:** biofilms, food industry, food microbiology, food safety

## Abstract

Biofilms, present as microorganisms and surviving on surfaces, can increase food cross-contamination, leading to changes in the food industry’s cleaning and disinfection dynamics. Biofilm is an association of microorganisms that is irreversibly linked with a surface, contained in an extracellular polymeric substance matrix, which poses a formidable challenge for food industries. To avoid biofilms from forming, and to eliminate them from reversible attachment and irreversible stages, where attached microorganisms improve surface adhesion, a strong disinfectant is required to eliminate bacterial attachments. This review paper tackles biofilm problems from all perspectives, including biofilm-forming pathogens in the food industry, disinfectant resistance of biofilm, and identification methods. As biofilms are largely responsible for food spoilage and outbreaks, they are also considered responsible for damage to food processing equipment. Hence the need to gain good knowledge about all of the factors favouring their development or growth, such as the attachment surface, food matrix components, environmental conditions, the bacterial cells involved, and electrostatic charging of surfaces. Overall, this review study shows the real threat of biofilms in the food industry due to the resistance of disinfectants and the mechanisms developed for their survival, including the intercellular signalling system, the cyclic nucleotide second messenger, and biofilm-associated proteins.

## 1. Introduction

Typically, bacteria bind to surfaces and form spatially structured communities inside a self-produced matrix, which consist of extracellular polymeric substances (EPS) known as biofilms [1,2]. Biofilms imply major challenges for the food industry because they allow bacteria to bind to a range of surfaces, including rubber, polypropylene, plastic, glass, stainless steel, and even food products, within just a few minutes, which is followed by mature biofilms developing within a few days (or even hours) [3].

**Figure 1:**
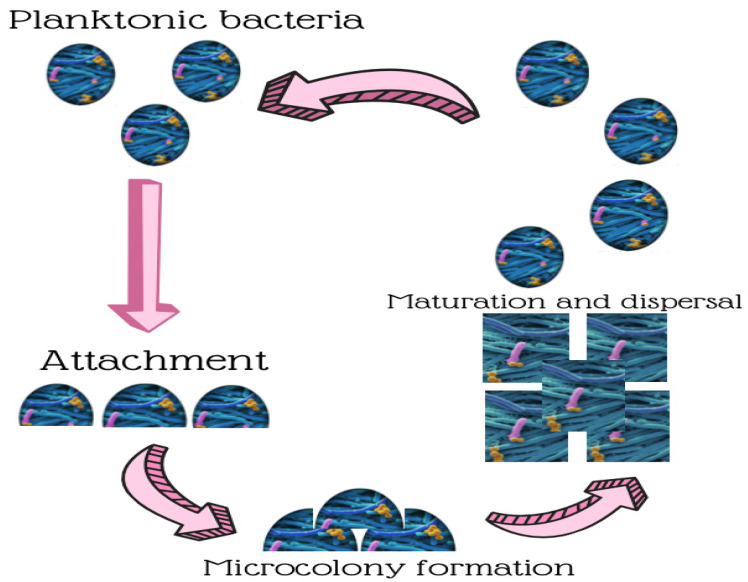
Biofilm formation and development stages.

**Figure 2:**
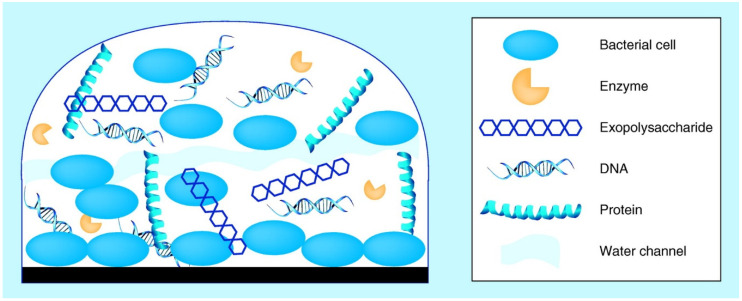
Biofilm structure in the growth and maturation stages [20].

Since ancient times, this sessile life form has been followed as an excellent survival technique for microorganisms, given the protective barrier generated and physiological changes made by the biofilm matrix, while it fights against the adverse environmental circumstances faced typically by bacteria in man-made and natural settings, even in food-processing facilities [4,5]. Hence, biofilms are believed responsible for damaged equipment, more expensive energy costs, outbreaks, and food spoilage [5,6,7,8]. Biofilms have become more robust to disinfections in many wide-ranging food industries, such as processing seafood, brewing, dairy processing, and meat and poultry processing [9] There is compelling evidence for biofilm lifestyle making them more resilient to antimicrobial agents, particularly compared to planktonic cells (Figure 1 and Figure 2). This entails having to remove them from surfaces of food processing plants, which poses a massive task [10,11,12].

Microbiological surface management is relevant for assessing and making decisions as to whether residual microbial species are found at a suitable level, and if harmful microorganisms are removed. The obtained results will allow criteria to be set, such as how to clean surfaces and food product quality [13,14].

Sensory tests that involve visually inspecting surfaces with good lighting, smelling unpleasant odours, and feeling encrusted or greasy surfaces are run as a process regulation to instantly overcome visible sanitation defects, while microbiological evaluations are often made to guarantee consistency with microbial standards and to make improvements to sanitation procedures [15]. The fact that visual inspection cannot coincide with bacterial counts has been well-documented [16]. The hygienic conditions of food-contact surfaces must be properly examined for all of the above-cited purposes. Lack of convergence between the various approaches followed to detect and quantify biofilms does, however, make it more difficult for the food industry to locate the most effective ones [17]. The Hazard Analysis and Critical Control Points (HACCP) system and good manufacturing practices have been developed to regulate food safety and quality. Bacterial biofilms are not directly mentioned in the HACCP system employed on food processing facilities. Hence, an updated HACCP system that contemplates evaluating biofilms in food environments, and establishes an apt sanitation plan, is expected to provide much clearer contamination information, and to facilitate production in the food industry’s biofilm-free processing systems [18]. The importance and impact of biofilms on the food industry have become clear in several works where the cross-contamination is common among these food products, with a wide range of pathogens, including *Listeria monocytogenes*, *Yersinia enterocolitica*, *Campylobacter jejuni, Salmonella* spp., *Staphylococcus* spp., *Bacillus cereus*, and *Echerichia coli* O157:H7 [19]. 

The main objectives of this review were to identify the most important biofilm examples in the food industry and to present methods to visualise in situ biofilm production, how to avoid this production, and methods to remove biofilms. This study focused on the microbial biofilms that affect the food industry and provides an overview of their importance in cross-contamination when food comes into contact with surfaces. Although going into detail in each discipline, specific to microbiology for biofilm isolation and identification, is not the object of this work, it contributes new knowledge about techniques to control and eradicate biofilms in the food industry from food safety and quality perspectives.

## 2. Biofilm Development in Food Processing Environments

Modern food processing lines are a suitable environment for biofilms to form on food contact surfaces, primarily due to manufacturing plants’ complexity, long production periods, mass product generation, and large biofilm growth areas [21]. Many food-borne bacteria may, therefore, bind to the contact surfaces present in these areas, which could contribute to increase the risk of bacterial food-borne diseases. By way of example, 80% of bacterial infections in the USA are believed to be related specifically to food-borne pathogens in biofilms [9].

Mixed-species biofilm production is extremely dynamic and depends on the attachment surface’s characteristics [22], food matrix components [23], environmental conditions [24], and involved bacterial cells [8,25].

Attachment surface properties, such as hydrophobicity, electrostatic charging, interface roughness, and topography impact biofilm formation and, thus, affect the overall hygiene status of the surface [22,26]. Nevertheless, the precise consequence of some parameters vastly varies under specific laboratory conditions. Some experiments have revealed that bacterial attachment is more likely to happen on rougher surfaces [22,27], while others have found no association between roughness and bacterial attachment [28]. Hydrophobic surfaces tend to attract more bacteria, but studies that have tested the hydrophobicity effect present opposing results [29,30], and other experiments indicate that hydrophilic surfaces enable more bacterial adherence than hydrophobic equivalents [27,28]. The fact that clear results are lacking might lie in the various methods and bacterial strains employed, and in overall attachment likely being established for several reasons. The most popular food contact material in the food industry is stainless steel type 304 because it is chemically inert, easy to clean, and extremely corrosion-resistant at a range of processing temperatures. Given its continuous usage, this material’s topography typically displays crevices and cracks that protect bacteria from sanitising treatments and mechanical cleaning methods.

The food matrix components in food processing environments also influence bacterial attachment [31]; e.g., food waste, such as milk and meat exudates enriched in fats, proteins, and carbohydrates, facilitate microorganism growth and multiplication, and favour dual-species biofilm development by *E. Coli* and *Staphylococcus aureus* [32]. Milk lactose improves biofilm production by both *Bacillus subtilis,* by activating the LuxS-mediated quorum-sensing system [33], and *S. aureus* through intercellular polysaccharide adhesion development [34]. Improved biofilm production by *Geobacillus* spp. in milk results in high concentrations of free Ca^2+^ and Mg^2+^ [35].

Microbial cell properties, especially hydrophobicity, cellular membrane components (e.g., protein and lipopolysaccharide), appendages (e.g., pili, flagella, fimbriae) and bacteria-secreted EPS, also play a key role in stimulating biofilm production [22]. Fluctuations in biofilm-forming capability among species or strains of different genotypes and serotypes have been identified, which reveals the evolution of enhanced biofilm formation from various genetic backgrounds [8,36]. Similar species can also impact one another in a mixed microbial community, which culminates in the co-colonisation of certain species.

## 3. Examples of the Most Relevant Biofilms in the Food Industry

In the food industry, biofilm-forming species appear in factory environments and can be pathogenic to humans because they develop biofilm structures. The processing environments of the food industry, e.g., wood, glass, stainless steel, polyethylene, rubber, polypropylene, etc., act as artificial substrates for these pathogens [37,38]. The characteristics of the bacterial growth form on food in a processing environment involve different behaviours when considering cleaning and disinfection processes. Controlling biofilm formations in the food industry can prove difficult when having to decide the right strategy.

Examples of these relevant biofilm-forming pathogens for the food industry are briefly described in Table 1. 

### 3.1. Bacillus Cereus

*Bacillus cereus* is a Gram-positive anaerobic or facultative anaerobic spore-forming bacterium that can grow in various environments at wide-ranging temperatures (4 °C–50 °C). It is resistant to chemicals, heat treatment, and radiation [39]. *B. cereus* is a frequently isolated soil inhabitant from food and food products, such as rice, dairy products, vegetables and meat. It secretes toxins that can cause sickness and diarrhoea symptoms in humans.

*B. cereus* is responsible for biofilm formation on food contact surfaces, such as stainless steel pipes, conveyor belts and storage tanks. It can also form floating or immersed biofilms, which can secrete a vast array of bacteriocins, metabolites, surfactants, as well as enzymes, such as proteases and lipases, in biofilms, which can affect food sensorial qualities [40]. Motility by bacterial flagella confers access to suitable biofilm formation surfaces, and is required for biofilms to spread on non-colonised surfaces. However, *B. cereus* flagella have not been found to be directly involved in adhesion to glass surfaces, but can play a key role in biofilm formation via their motility [55].

### 3.2. Campylobacter Jejuni

*Campylobacter* spp., mainly *C. jejuni*, are Gram-negative spiral, rod-shaped, or curved thermophilic and bipolar flagellated motile bacteria [41]. *C. jejuni*, also known as an anaerobic bacterium, can develop biofilms under both microaerophilic (5% O_2_ and 10% CO_2_) and aerobic (20% O_2_) conditions [56]. Despite it being a fastidious organism, *C. jejuni* can survive outside the avian intestinal tract before it reaches a human host. A range of environmental elements initiates the formation of biofilms, which are then affected by a set of intrinsic factors [57]. The European Union One Health 2018 Zoonoses Report classifies *C. jejuni* as an opportunistic pathogen that is believed to be the causative agent of most bacterial gastroenteritis cases, and has been regarded as a common commensal of food animals and poultry, with turkeys and hens in particular [42]. When the preparation and processing areas of food products or water become contaminated, such as unpasteurised milk, *C. jejuni* reaches the human host by infecting and colonising the gastrointestinal tract to cause disease [43].

### 3.3. Enterohaemorrhagic Escherichia coli (EHEC)

*Escherichia coli* is a Gram-negative and rod-shaped bacterium. Most *E. coli* strains form part of human intestinal microbiota and pose no health problem. However, the virulence types of *E. coli* include enterotoxigenic (ETEC), enteroinvasive (EIEC), enteropathogenic (EPEC), and Vero cytotoxigenic (VTEC). O157:H7 EHEC is the most frequent serotype associated with EHEC infections in humans in the USA [58]. Widespread *E. coli* dissemination in natural environments is, to a great extent, due to its ability to grow as a biofilm. It is worth considering that several *E. coli* strains may cause disease in humans, and that Enterohaemorrhagic *E. coli* (EHEC) strains are the most relevant for the food industry. EHEC serotype O157:H7 is the human pathogen responsible for bloody diarrhoea outbreaks and haemolytic uremic syndrome (HUS) worldwide. They can be transmitted by raw milk, drinking water or fresh meat, fruit, and vegetables; e.g., melons, tomatoes, parsley, coriander, spinach, lettuce, etc. [44].

*E. coli* can employ pili, flagella and membrane proteins to initiate attachment to inanimate surfaces when flagella are lost after attachment and bacteria start producing an extracellular polymeric substance (EPS) that helps to confer bacteria better resistance to disinfectants [59]. There are reports indicating that although EHEC can form biofilms on different food industry surfaces, neither an effective means to prevent EHEC biofilm formation nor an effective treatment for its infections exists because antibiotic treatment tends to increase the risk of haemolytic-uremic syndrome and kidney failure [60].

### 3.4. Listeria Monocytogenes

*Listeria monocytogenes* is a Gram-positive bacterium and a ubiquitous food-borne pathogen that can appear in soil, food, and water. Its ingestion can result in abortions in pregnant women, and other serious complications in the elderly and children. The pathogen can be transmitted to several food types, such as dairy products, seafood, meat, fruit, ready-to-eat meals, ice cream, soft cheeses, unpasteurised milk, frozen vegetables, candied apples, and poultry [45,46], but it is not known to be resistant to pasteurisation treatments [61]. The pathogen proliferates at low temperature, and is able to form pure culture biofilms or grow in multispecies biofilms [62]. *L. monocytogenes* can survive under acidic conditions for lengthy periods and can form biofilms that grow without oxygen. Its numbers are likely to rise or lower in biofilms depending on the competing microbes present [63].

Given the presence of pili, flagella and membrane proteins, prevalent *L. monocytogenes* strains possess good adhesion ability in food processing environments [64].

### 3.5. Salmonella Enterica

*Salmonella enterica* is a Gram-negative, rod-shaped, flagellate and facultative aerobic bacterium, and a species of the genus *Salmonella* [65].

It can cause gastroenteritis or septicaemia (in some serovars) [66]. *Salmonella* spp. express proteinaceous extracellular fibres known as curli, which are involved in surface and cell-cell contacts, and in promoting community behaviour and host colonisation [67]. Besides curli, different fimbrial adhesins have been identified with biofilm formation implications that are serotype-dependent [40]. *S. enterica serovar Enteritidis* is the most frequent serotype to cause fever, vomiting, nausea diarrhoea, and abdominal pain as main symptoms [47]. Poultry meat is a frequent reservoir for these bacteria in processed food, whose importance as a food pathogen has been demonstrated by the fact that *S. enterica* biofilm formation on food surfaces was the first reported case in 1966 to possess complex multicellular structures [48]. 

When contaminating a food pipeline biofilm, *S. enterica* may cause massive outbreaks, and even death in infants and the elderly. It can grow on stainless steel surfaces to form a three-dimensional (3D) structure with several call layers of different morphologies depending on available nutrients, such as the reticular shaped ones generated when cultured on tryptic soy broth (TSB) medium [68].

### 3.6. Staphylococcus Aureus

*Staphylococcus aureus* is a Gram-positive, non-spore-forming, non-motile, facultative anaerobic bacterium capable of producing enterotoxins from 10–46 °C. *S. aureus* can multiply on the skin and mucous membranes of food handlers, and can become a major issue in food factories [49]. These enterotoxins are heat-stable and can be secreted during *S. aureus* growth in foods contaminated by food handlers. The bacterium grows well in high salt- or sugar-content foods with little water activity. The foods frequently implicated in Staphylococcal food-borne disease are meat and meat products, poultry and egg products, milk and dairy products, bakery products, salads, and particularly cream-filled cakes and pastries and sandwich fillings [50]. *S. aureus* is known for its numerous enteric toxins. These enterotoxins bind to class II MHC (major histocompatibility complex) in T-cells, which results in their activation that can lead to acute toxic shock with sickness and diarrhoea [69].

### 3.7. Pseudomonas spp.

*Pseudomonas* is a heterotrophic, motile, Gram-negative rod-shaped bacterium. Pseudomonads are generally ubiquitous psychrotrophic spoilage organisms that are often found in food processing environments, including floors and drains, and also on fruit, vegetables, and meat surfaces, and in low-acid dairy products [17,62]. The extracellular filamentous appendages produced by motile microorganisms result in both the attachment process and the interaction with surfaces in different ways. Flagella and pili have been thoroughly studied [70].

When biofilms develop and their regulation by quorum sensing is considered, *Pseudomonas aeruginosa* can be taken as a model organism [71], which is about 1–5 µm long and 0.5–1.0 µm wide. A facultative aerobe grows via aerobic and anaerobic respiration with nitrate as the terminal electron acceptor [71].

*Pseudomonas* spp. produce huge amounts of EPS and are known to attach and form biofilms on stainless steel surfaces. They can co-exist with other pathogens in biofilms to form multispecies biofilms, which make them more resistant and stable [62]. These biofilms can be accompanied by a distinct blue discolouration (pyocyanin) on fresh cheese produced by *P. fluorescens* [72].

### 3.8. Geobacillus stearothermophilus

*Geobacillus stearothermophilus* is a Gram-positive, thermophilic, aerobic, or facultative anaerobic bacterium [73]. Thermophiles, such as *G. Stearothermophilus,* formerly known as *Bacillus stearothermophilus,* can attach to stainless steel surfaces on processing lines in evaporators and plate heat exchangers, which allows them to grow and produce biofilms, which implies the potential release of single cells or aggregates of cells into the final dry product [74]. *B. stearothermophilus* are able to form biofilms on clean stainless steel surfaces and to release bacteria into milk during dairy industry processing [75]. The above-cited authors observed that the conditions for a biofilm in a laminar flow milk system were more adequate for the growth of spore-forming bacteria, which are thermophilic. Their growth as a culture medium in milk is quite difficult [75].

### 3.9. Anoxybacillus flavithermus

*Anoxybacillus flavithermus* is another Gram-positive, thermophilic, and spore-forming organism that is facultatively anaerobic and non-pathogenic [76]. *A. flavithermus* is a potential contaminant of dairy products, and poses a problem for the milk powder processing industry, as high levels will reduce milk powder acceptability for both local and international markets [77]. *A. flavithermus* spores are very heat-resistant and their vegetative cells can grow at temperatures up to 65 °C with a significant increase in bacterial adhesion on stainless steel surfaces in the presence of skimmed milk. This indicates that milk positively influences these species’ biofilm formation [78]. In the dairy industry, the commonest biofilm-forming isolates are thermophilic genera [79]. In many parts of the world, *A. flavithermus* and *G. stearothermophilus* are regarded as the most dominant thermophilic microbial contaminants of milk powders [78].

### 3.10. Pectinatus spp.

*Pectinatus* is Gram-negative, non-spore-forming, and anaerobic bacteria that have been linked with a high concentration of biofilms in breweries due to sanitation problems [80]. Spoilage bacteria were first isolated from a brewery in the USA in unpasteurised beer stored at 30 °C [81]. *P. cerevisiiphilus* have also been isolated from many breweries in Germany, Spain, Norway, Japan, the Netherlands, Sweden, and France [80].

### 3.11. Synergistic Pathogens

A combination of several pathogens can synergistically interact to form biofilms in the food industry. In food-processing environments, bacteria are able to exist as multispecies biofilms, from where both spoilage and pathogenic bacteria can contaminate food [82]. For instance in the fishing industry, fresh fish products can suffer from biofilm formation by mixed pathogenic species (*Aeromonas hydrophila*, *L. monocytogenes*, *S. enterica,* or *Vibrio* spp.), which can imply significant health and economic issues [83]. Synergistic interactions have been observed in a fresh-cut produce processing plant, where *E. coli* interacted with *Burkholderia caryophylli* and *Ralstonia insidiosa* to form mixed biofilms. Acylhomoserine lactones (AHLs) can control biofilm formation in synergistic interactions among mixed species. Interference of AHLs is manifested by AHL lactonases and acylases, both of which are present in Gram-positive and Gram-negative bacteria [60].

Bacteria use quorum sensing to coordinate biofilm production and dispersion, when bacteria attach to a biotic or abiotic surface, and cell-to-cell attachment engages in communication via a quorum sensing (QS)-based extracellular cell signalling system [84]. The importance of cell signalling for bacterial biofilm formation has been further confirmed by the control of exopolysaccharide synthesis by quorum-sensing signals, as in *Vibrio cholera* [85].

Synergistic pathogens are found in several works, where biofilm levels of the four-species consortia have been further examined and compared to the biofilm production levels of each isolate under monospecies conditions. They have revealed that *P. aeruginosa* and *A. junii* isolated from different samples to contribute as best biofilm producers, including poor or non-biofilm-producing isolates, which increases the overall biofilm formation in the included consortia [86]. Several authors [87] have found positive synergistics in other studies by investigating mixed species of biofilms, such as *Candida albicans.*

In food industries, biofilm-related effects (pathogenicity, corrosion of metal surfaces, and alteration to organoleptic properties due to the secretion of proteases or lipases) are critically important. For example, in the dairy industry several processes and structures (pipelines, raw milk tanks, butter centrifuges, pasteurisers, cheese tanks, packing tools) can act as surface substrates for biofilm formation at different temperatures and involve several mixed colonising species. Thus, it is essential that accurate methods to visualise biofilms in situ be set up to avoid contamination and to ensure food safety in the food industry.

## 4. Biofilm Control and Elimination

It is well-known that biofilm bacteria present a distinct phenotype with a genotype as regards gene transcription and growth rates under very particular conditions that differ from planktonic conditions [88]. Biofilms are capable of adhering to a very wide diversity of surfaces with distinct biotic and abiotic compositions, including human tissue and medical devices. Once biofilms form, they are a major threat because they cause infectious diseases and economic loss. In the 1940s, several authors produced further research works into biofilm evolution and surface relations for marine microorganisms [89] and seawater [90]. Nevertheless, marked progress has been made given the incorporation of the electron microscope, which allows high-resolution photomicroscopy at much higher magnifications than light microscopy [85]. Indeed, the most revealing discovery of the relation with biofilm elimination was a description of its structure, the surrounding matrix material, and the cells enclosed in these biofilms were polysaccharides, as by special stains revealed [85]. Doubtlessly, disinfectants have proven more efficient in fighting against biofilms since 1973, while Characklis (1973) [91] showed marked persistence and resistance to disinfectants, such as chlorine.

### 4.1. Biofilm Elimination in the Food Industry

Biofilm formation has been investigated in food industry and hospital environments. Perhaps, in the research conducted, hospitals, to eliminate biofilms, have been more successful, thanks to the easier applications and special surface compositions (antibiofilm activity) in medical surroundings, such as implants, prostheses, tools, and surfaces for operating theatres.

To date, many efforts have been made to reduce biofilm formation on food industry surfaces, but those works were based mainly on new disinfectants with different efficacies. These results have improved in line with specific mechanisms for initial surface attachment, developing a group structure and ecosystem, and detachments [75] with dissimilar results.

Nowadays, disinfectants are the best ally to eliminate biofilms. However, other research fields, such as the composition of surfaces for materials to prevent bacterial adhesion and developing phages to combat biofilm-forming bacteria, have obtained favourable results.

Doubtlessly, most research works have focused on the bacteriological biofilm, without discussing the hypothesis of filamentous fungi being responsible for biofilm formation. Several authors [92,93] support this theory, where the presence of Aspergillus fumigatus has been presented as biofilm-responsible. In this case, the marked similitude of bacteriological and filamentous fungi biofilms is based on morphological changes, the presence of an extracellular polymeric matrix, differential gene expression, and distinct sensitivity to antifungal drugs compared to diffuse or loosely associated (planktonic) colonies [94,95].

Before we go on to explain several factors that could influence bacterial adhesion and biofilm formation, we should bear the food industry’s hygiene design in mind. In order to prevent microorganisms entering food production, factories, and the employed hygienic equipment should be designed to limit microorganisms from accessing. Aseptic equipment must be isolated from microorganisms and foreign particulates. To prevent microorganism growth, equipment should be designed to prevent any areas where microorganisms can harbour and grow, along with gaps, crevices, and dead areas. This is also important during production, when microorganisms can grow very quickly under favourable conditions [96].

According to such premises, food companies have the capacity to apply innovation to design the industry and its equipment. In both the USA and the European Union (EU), the trend in regulations in this field is not so much command and control by government regulators, but lies more in self-determination by the food industry. In particular, hazard analysis and critical control points (HACCP) systems provide the skills to replace detailed regulatory requirements with the overall goals to be fulfilled [97]. The Food and Drug Administration (FDA) and the United States Department of Agriculture (USDA) Food Safety Inspection Service (FSIS) share primary responsibility for regulating food safety in the USA. One example is the recommendation for equipment and process controls: “Seams on food-contact surfaces shall be smoothly bonded or maintained to reduce accumulation of food particles, dirt, and organic matter and thus minimise the opportunity for growth of microorganisms” [85]. 

European Union (EU) statutory instruments include EC regulation no. 852/2004 (Hygiene of Foodstuffs) and EC regulation no. 853/2004 (Specific Rules Food of Animal Origin), which expect food manufacturers to control food safety risks by HACCP systems.

In short, the performance of a cleaning and disinfection programme (CDP) to avoid biofilm formation should start by aptly designing the hygiene of equipment, surfaces, and devices. Today the CDP is a proven effective measure in fighting against biofilms.

### 4.2. Factors Associated with Bacteria

Several factors associated with surfaces or bacteria can influence adhesion from the planktonic phase and biofilm evolution, such as:

Surfaces:

- Surface charge: the adhesion sequence can be influenced by the particle surface charge in combination with the electrode’s surface charge.
- Hydrophobicity: adsorption of surface-active organics influences the surface’s hydrophobic or hydrophilic character, and changes surface tension [98].
- Temperature: temperature and contact time impact the bacterial adhesion and biofilm formation process, and increasing the formulation of mathematical models is necessary to assess how both factors and their interactions influence the process [99].
- Presence of substrates: adsorption of electrolyte components and the particle surface are equally important. Adsorption of surface-active organics impacts the surface’s hydrophobic or hydrophilic character, and also changes surface tension [98].

Bacterial cellular surface components:

Hydrophobic interactions tend to increase with the enhanced non-polar nature of one involved surface or more, and most bacteria are negatively charged [85]. This means that the cell surface’s hydrophobicity is a relevant factor during adhesion.

- Fimbria, pili, and flagella: fimbriae, non-flagellar appendages other than those implicated in the transfer of viral or bacterial nucleic acids (known as pili) are responsible for cell surface hydrophobicity. Most investigated fimbriae contain a high proportion of hydrophobic amino acid residues [100]. Fimbriae include adhesins that attach to some sort of substratum so that bacteria can withstand shear forces and obtain nutrients. Therefore, fimbriae play a role in cell surface hydrophobicity and attachment, presumably by overcoming the initial electrostatic repulsion barrier between the cell and the substratum [101].

Studies’ responses surface methods are used to not only develop and optimise models for food processing systems and operations, but also to better elucidate bacterial adhesion and biofilm formation processes. The response surface method provides valuable information to help decision making about disinfection and cleaning procedures for the utensils, equipment and containers employed in the food industry [102]. Hence, surfaces and materials of equipment, plus floors and walls, also impact biofilms, along with dead spaces, crevices, porous and rough material surfaces, which must be eliminated to avoid biofilm formation [12].

The most adopted strategies for controlling biofilms are sanitation procedures that combine detergents and disinfectants. Alkaline detergent eliminates organic and inorganic acid detergent waste from surfaces, while disinfectants reduce spoilage microorganisms, and diminish or eliminate pathogens, to safe levels [12]. Enzymatic detergents have replaced traditional alkaline and acid detergents, because enzymes (proteases, lipases, amylases) can remove biofilms in the food industry [102] as enzymes reduce the physical integrity of PS by weakening the structural bonds of the lipids, proteins, and carbohydrates that form its structure [103]. Other advantages over detergents include low-toxicity and biodegradability, but application costs and requirements (temperature, time) are higher than detergents. This is why several detergent manufacturers have marketed a synergetic combination of enzymes, chelating agents, surfactants, and solvents.

### 4.3. Disinfectants and Biofilm Resistance

The most widely used disinfectants in the food industry’s disinfection programmes are quaternary ammonium compounds (QAC), amphoteric compounds, hypochlorites, peroxides (peracetic acid and hydrogen peroxide) [15], aldehydes (formaldehyde, glutaraldehyde, paraformaldehyde), and phenolics. This product list remains unchanged after 18 years. Today, alkyl amines, chlorine dioxide and quaternary ammonium blends are incorporated into disinfection programmes. Besides these, alcohols, phenolic compounds, aldehydes, and chlorhexidine are also resorted to, but mostly in health services. In the food processing industry, disinfectants can remain on surfaces for longer due to microorganisms’ prolonged exposure to the employed disinfectant, which improves their efficacy [104].

1. Sodium hypochlorite (NaOCI):

It is one of the most widespread disinfectants in the food industry despite its disadvantages and the growing use of new products on the market. Its desirable reaction produces both hypochlorous acid (HOCl) and the hypochlorite ion (-OCl), which are strong oxidising agents that eliminate cells given their ability to cross the cell membrane, and to oxidise the sulfhydryl groups of certain enzymes participating on the glycolytic pathway [97]. It has been described to react with wide-ranging biological molecules, such as proteins [105], amino acids [106], lipids [107], peptides [108], and DNA [109] under physiological pH conditions [110].

However, sodium hypochlorite may be affected by organic matter because free chlorine might react with natural organic matter and be converted into inorganic chloramines to generate trihalomethanes, by reducing antimicrobial activity against biofilms [111], and it is known to be less reactive than free chlorine. Peracetic acid is reported as being the most effective sanitiser against biofilms because it is a strong oxidising agent that does not interact with organic matter waste [112,113].

2. Quaternary ammonium:

QACs, such as benzalkonium chloride, cetrimide, didecyldimethylammonium chloride, and cetylpyridinium chloride, are cationic detergents (surfactants or surface-active agents). They reduce surface tension and form micelles to lead to dispersion in a liquid. This property is resourceful for removing microorganisms. They are membrane-active agents that interact with not only the cytoplasmic membrane of bacteria, but with the plasma membrane of yeast. Their hydrophobic activity also makes them effective against lipid-containing viruses. QACs interact with intracellular targets and bind to DNA [114]. However, their efficacy is still questioned given the appearance of relatively high resistance to *Listeria monocytogenes* (10%), *Staphylococcus* spp. (13%), and *Pseudomonas* spp. (30%), and lower resistance to lactic acid the bacteria (1.5%) and coliforms (1%) isolated from food and the food processing industry [115].

3. Peracetic acid:

In the last decade, peracetic acid (PAA) has been widely used by the food industry in water and wastewater treatment, even in paper machines [116], to control biofilms. Its antimicrobial effect is probably due to the oxidation of thiol groups in proteins, disruption of membranes [117], or damage to bases in DNA [118]. Its use has been shown to increase the sensitivity of bacterial spores to heat [119]. The efficacy and environmental safety of peracetic acid make it an attractive disinfecting agent for industrial use. 

Bacterial regrowth after oxidant treatment (peracetic acid and free chlorine) depends on the absence or presence of organic matter. The oxidation-reduction (redox) potential of PAA (1.385 V vs. standard hydrogen electrode or standard hydrogen electrode (SHE) for the redox couple of CH_3_COOOH(aq)/(CH_3_COO^−^_(aq)_ + H_2_O_(l)_)) comes close to that of free chlorine (1.288 V vs. SHE for the redox couple of HOCl_(aq)_/Cl^−^_(aq)_) under biochemical standard state conditions (pH 7.0, 25 °C, 101.325 Pa) [120]. For this reason, acid peracetic and free chlorine may have similar efficiencies in preventing planktonic bacteria regrowth in the absence of organic matter. As PAA reacts with organic matter more slowly than free chlorine, its self-decomposition is slower [121]. 

#### Resistance to Disinfectants

Bacterial resistance to disinfectants in the planktonic phase can hardly be compared to biofilm resistance. Yet several studies have shown contrary results to widespread belief, such as the existence of wide interspecific variability of resistance to disinfectants. Gram-positive strains generally appear to better resist than Gram-negative strains. This resistance is also variable among strains of the same species [122].

Given the growing interest in knowing biofilm resistance to chlorine, quaternary ammonium and peracetic acid, many studies have been conducted [123]. The bacteria in mature biofilms are 10- to 1000-fold more resistant to antibiotics than the bacteria in the planktonic phase [124], and this resistance appears against biocides. However, this natural resistance is still unknown, and probably depends on many factors, mainly of structural biofilm barriers and genetic factors for adaptation. To explain this resistance, several authors [125] have suggested three possible causes by three hypotheses: the first is based on the slow or incomplete diffusion of antibiotics into inner biofilm layers. The second lies in the changes taking place in the biofilm microenvironment as some biofilm bacteria fall into a slow growth state due to lack of nutrients or given the accumulation of harmful metabolites and, therefore, survive [126]. Finally, the third hypothesis indicates a subpopulation of cells in the biofilm whose differentiation resembles the spore formation process. They have a unique and highly resistant phenotype to protect them from the effects of antibiotics, and are a biologically programmed response to the sessile life form of bacteria [127].

In addition, aquatic fauna is less affected by PAA than by chlorine [128]. Therefore, PAA is considered a green alternative to chlorine for disinfection purposes, and its disinfection performance is currently being investigated [120].

As previously mentioned, there are two very different situations for action against biofilm formation: the food industry and the health field. There is still a lot to do in both fields, but it is true that CDP is practically the only implementation in the food industry. The health field has witnessed much more progress: phages [129], aerosolisation [130], sonication brush [131], and metal ion solutions (silver, copper, platinum, gold, and palladium) [132].

In short, some authors [133] establish two strategies for fighting biofilms in the food industry: structural modification of surfaces or application of antibacterial or antibiofilm coatings [134]. Thus, several alternative products to classic disinfectants (chlorine, quaternary ammonium, etc.), such as, plant-derived antimicrobials (essential oils: orange-sorrel, lemon, lavender, chamomile, peppermint, oregano), with thymol and carvacrol being the compounds that display more significant antimicrobial action in shorter action times. 

### 4.4. Alternative Methods to Eliminate Biofilms

Phages: bacteriophages are specific “viruses” of microbial cells that are specific to the different serotypes or strains of microbial species, and are obligate parasites with a genetic parasitism [135]. Bacteriophages inject their DNA and force the cell to produce the bacteriophage genome and structures (e.g., capsid and tail). When phages are complete, they lyse cells, which means that bacteriophage infection can destroy the entire colony [136,137]. In the last few years, the FDA/USA approved preparing bacteriophages (LISTEX P100) to combat the direct presence of *L. monocytogenes* in foods [138,139]. 

Aerosolisation: a disinfection method with different disinfectants applied to working areas by pulverisation. Several authors [138] have shown its efficacy as a biofilm control method in the food industry and hospitals by using hydrogen peroxide [140], sodium hypochlorite, and peracetic acid [141].

Knowledge about the resistance mechanisms associated with biofilm evolution could be primordial to develop new actions or strategies by biocides and antibiotics [142], such as modified wound dressings with phyto-nanostructured coatings to prevent staphylococcal and pseudomonal biofilm development [143].

Involving bacterial adherence: in recent years, new biochemistry methods have been studied to prevent biofilm formation. The most efficient strategy would interfere with bacterial adherence, as this first step is paramount in biofilm formation, performed by the direct blockage of surface receptors [144] or by a non-specific strategy, which normally involves compounds with anti-adherence properties [145]. Another biofilm inhibition form would be to impede communication processes between bacteria to enter the biofilm by employing different natural or artificially synthesised compounds [146]. One example of this is *P. aeruginosa,* which uses quorum sensing for modular biofilm evolution, and proposes that agents are capable of blocking quorum sensing (QS), and could be useful for avoiding biofilm formation [144].

The role of the QS in biofilms includes controlling the cell-to-cell communicating system in response to small diffusible signal molecules, such as N-acyl-homoserine lactones (AHLs), produced by Gram-negative bacteria [147]. It has been shown to play crucial roles in biofilm formation by activating the transcription of related genes [148]. Specifically, different AHLs have been detected in biofilm reactors and several bacterial species have been identified to possess the capacity to produce AHLs [149]. Thus, AHLs-based QS has been widely reported to regulate many microbial behaviours, including EPS expression, nitrogen transformation, organic pollutant degradation and microbial community construction [150].

Current advances in nanoscience nanotechnology and nanosensors [151] have emerged for applications to detect microorganisms and biofilms [152] with high sensitivity and good spatial resolution on nanoscale scopes [153]. Recent works have been published on imaging approaches of biofilm microprocesses [154], in-situ surface-enhanced Raman Scattering (SERS) analyses, biofilm visualisation [155] and biosensors of bacteria in foods [156]. The enormous advantages and great potential observed in bioassays based on multifunctional optical nanosensors are promising to continue with a view to ensure and promote food safety and quality. From the detection targets perspective, QS detection might become a new biofilm research trend based on evidence that biofilm formation can be inhibited by blocking QS [151].

## 5. Biofilm Identification Techniques and Methods to Visualise Biofilms In Situ

### 5.1. General Aspects of Biofilm Study Techniques

Doubtlessly, biofilms can pose a major challenge in both clinical microbiology and hygiene food areas. In the latter area, several authors consider them a real threat. Currently, methods aim to analyse biofilm formation and development, which have not yet been standardised. Different methods have been followed to qualitatively and quantitatively evaluate biofilms, and each one is useful for estimating one peculiar biofilm lifestyle aspect [157]. Nevertheless, research to identify and acquire knowledge of biofilms has allowed distinct techniques to be developed and adapted from microbiology or cell histology. It is essential to evaluate biofilm formation for a sensitive, specific and reproducible methodology for biofilm quantification to become available. 

Different approaches classify the methods followed to detection biofilms on very distinct surfaces: (i) the simplest classification of methods is direct or indirect [17]. (ii) Rapid tests of hygienic control, and methods for microscopic, biomolecular, extracellular polymeric, physical, or chemical substances (EPS) are another possible classification [158]. (iii) A recent publication [159] only refers to the technology of the referred methods being classified as physics, physico-chemistry or chemistry, and recommends three effective approaches for testing biofilms: (a) observations by various microscopic methods with different view fields at the same point; (b) in-depth data analyses during microscopic image processing; (c) a combination study using atomic force microscopy (AFM) and chemical analysis. Perhaps this is the most advanced and appropriate methodology for biofilm analyses, where the detailed image of the surface will help to build a relation among the biofilm matrix, interactions and other factors like pH, surfaces [159]. However, there is another case in which bacterial species can help to reduce biofilms, where *Bacillus licheniformis* can express hydrolytic enzymes capable of reducing detrimental biofilms [160].

Therefore, depending on the set objectives, that is, what we wish to achieve with the biofilm, we should choose a technique according to our study. Not all techniques are suitable for a certain purpose, but might be compatible. Thus, some methods are suited for quantifying the biofilm matrix, while others are able to evaluate both living and dead cells, or exclusively quantify viable cells in biofilms. 

By considering the complexity and heterogeneity of the biofilm structure, the exact research objective should be set. The amount of EPS, the total number of bacterial cells embedded in biofilms, or the actual number of living bacteria in biofilms must be considered to be different targets that require distinct experimental approaches [157]. We should bear in mind that the biofilm volume is constituted mainly by an extracellular matrix (95–65% range), which is composed mostly of proteins (>2%), and other constituents, such as polysaccharides (1–2%), DNA molecules (<1%), RNA (<1%), ions (bound and free), and finally 97% water [161]. Thus, the biofilm research methodology should address the identification of bacteria and other matrix constituents.

In order to obtain a fundamental understanding of the formation and presence of bacterial biofilms, our analysis should include the detection of bacteria and the matrix. The most frequently followed methods to assess biofilm heterogeneity are direct microscopic imaging of the local biofilm morphology or microscopic measurements of local biofilm thickness [162]. For many applications, time-lapse microscopy with Confocal Laser Scanning Microscopy (CLSM) is an ideal tool for monitoring at a spatial resolution in the order of micrometres, and it allows the non-destructive study of biofilms by examining all layers at different depths. In this way, it is possible to reconstruct a three-dimensional structure [163]. Matrix detection can be achieved by a double-staining technique combined with CLSM, which allows the simultaneous imaging of bacterial cells and glycocalyx in biofilms [164]. 

### 5.2. Colorimetric Methods

#### 5.2.1. Evaluating the Biofilm Matrix

Staining the biofilms grown in microtiter plates wells is widely utilised by researchers to screen and compare biofilm formation by different bacteria or under various conditions [165]. Of the methods described in the literature, crystal violet (CAS number 931418 92 7) [166] is the most widespread for biofilm biomass quantification [167,168]. This basic dye binds negatively charged molecules and, thus, stains are able to dye both bacteria and the surrounding biofilm matrix. Acetic acid can be used as the extraction solvent and be measured by absorbance at 700–600 nm. Safranin staining can also be employed for biofilm biomass quantification [165,169], but results in lower optical densities than crystal violet staining and, therefore, may not be as sensitive to detect small amounts of biofilm [165].

Crystal violet staining tests the concentration of the dye incorporated into bacterial cell walls, and depends on cells’ integrity, but not on viability. However, other methods like ATP bioluminescence report the cell’s metabolic status and drops to undetectable limits within minutes after cell death. Resorting to both methods can provide supplementary information on the cell exposed to disinfectant. The results can indicate that, despite the drastic drop in viable cell numbers in the biofilm after disinfectant treatment, a significant number of intact cells, or cellular debris, may still be capable of retaining the dye. This observation leads to the question about the reliability of crystal violet staining as a method to monitor biofilm disinfection [170].

Another colorimetric method for living cells is fluorescein diacetate (CAS number 596 09 8), which employs a useful live-cell fluorescent stain that is hydrolysed to fluorescent fluorescein in live cells. The signal can be spectrophotometrically measured. This is suitable for cell viability assays with intact membranes as dead cells are unable to metabolise fluorescein diacetate. Thus, there is no fluorescent signal [157]. 

#### 5.2.2. Cell Staining

Visualising a cell with fluorescent compounds provides a wide variety of information to analyse cell functions. Various activities and cell structures can be targeted for staining with fluorescent compounds [171]. These cell components are mostly cell membranes, nucleotides, and proteins. The stain can pass to cells depending on the molecule charge, hydrophobicity, or reactivity. Thus, small neutral and positively charged fluorescent compounds can normally reach mitochondria for dyeing. Negatively charged molecules cannot pass through viable cell membranes. Ester is a suitable functional group for staining viable cells because it can pass through viable cell membranes, where it is hydrolysed by cellular esterases into a negatively charged compound [171].

Other complementary techniques can be run to examine the performances of advanced microscopic techniques employed to study microbial biofilms (i.e., confocal laser scanning microscopy, mass spectrometry, electron microscopy, Raman spectroscopy) [157].

Spectrofluorometric assays for the quantification of biofilms of gram-negative and gram-positive bacteria is a method that utilises the specific binding of the wheat germ agglutinin-Alexa Fluor 488 conjugate (WGA) to N-acetylglucosamine in biofilms [172]. This lectin conjugate also binds to N-acetylneuraminic acid on the peptidoglycan layer of gram-positive bacteria. WGA specifically binds to polysaccharide adhesin (poly N-acetylglucosamine), which is involved in biofilm formation by both gram-positive and gram-negative bacteria. Burton et al. [172] compared the colorimetric assay with the spectrofluorometric assay, whose results revealed that WGA staining may be a more specific means of *E. coli* and *Staphylococcus epidermidis* biofilm detection and quantification.

#### 5.2.3. LIVE/DEAD

**Figure 3:**
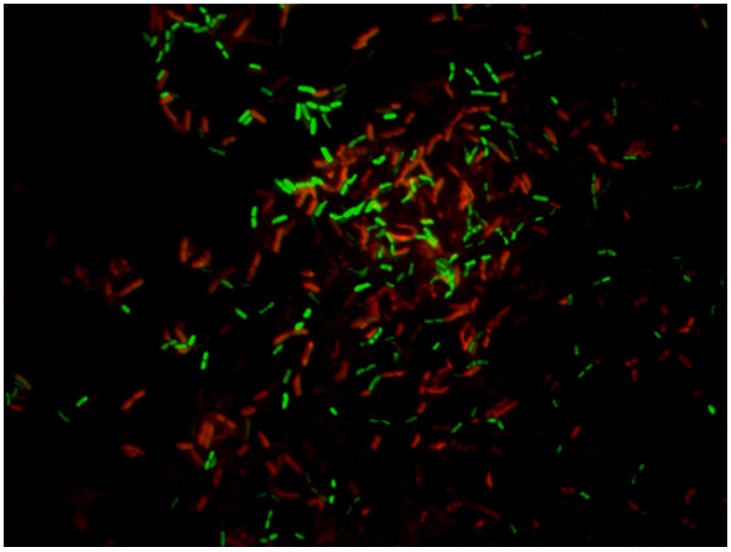
Planktonic state of *Pseudomonas fluorescens* stained with LIVE/DEAD (SYTO 9 and propidium iodine) after treatment with sodium hypochlorite (500 ppm) for 15 min. Viable bacteria (green) and damaged bacteria (red). Magnification ×100.

**Figure 4:**
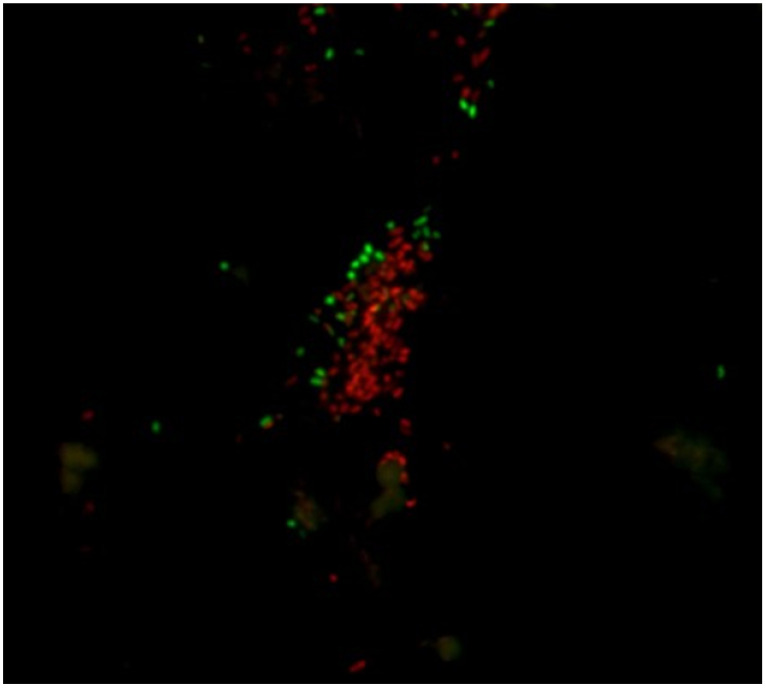
Planktonic state of *Pseudomonas fluorescens* stained with LIVE/DEAD (SYTO 9 and propidium iodine) after treatment with peracetic acid (250 ppm) for 15 min. Viable bacteria (green) and damaged bacteria (red). Magnification ×100.

**Figure 5:**
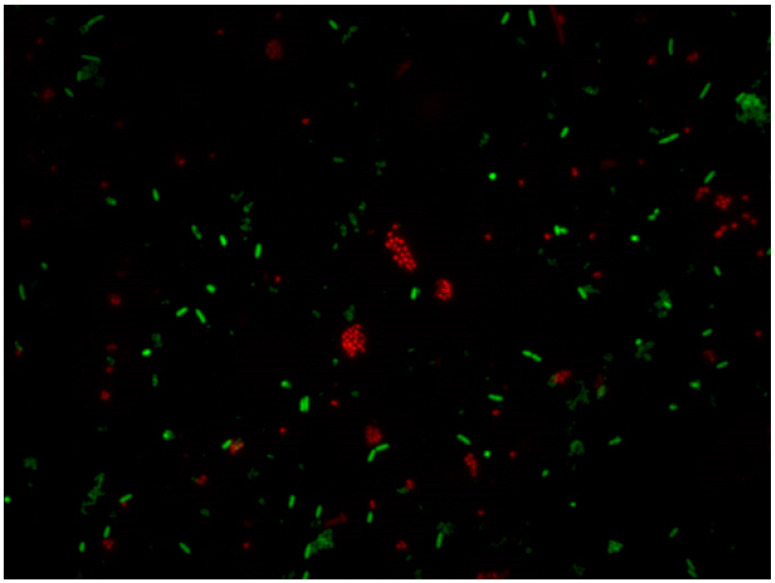
Planktonic state of *Pseudomonas fluorescens* stained with LIVE/DEAD (SYTO 9 and propidium iodine) after treatment with Sodium hypochlorite (350 ppm) for 15 min. Viable bacteria (green) and damaged bacteria (red). Magnification ×100.

This method is based on employing two different nucleic acid binding stains. The first dye is green fluorescent (Syto9, λ_ex_ 486 nm and λ_em_ 501 nm), which is able to cross all bacterial membranes and bind to the DNA of both Gram-positive and Gram-negative bacteria. The second dye is red-fluorescent (propidium-iodide (PI), CAS number 25535-16-4, λ_ex_ 530 nm and λ_em_ 620 nm), which crosses only damaged bacterial membranes. Stained samples are observed under a fluorescent optical microscopy to evaluate live and dead bacterial populations (see Figure 3, Figure 4 and Figure 5). In fact, live bacteria fluoresce in green and dead bacteria fluoresce in orange/red [173]. The efficiency of both stains is conditioned by some factors, such as the reagent’s binding affinity to cells [169], physiological cell state [174], reagent concentration [175], and temperature and incubation time [176].

Both stains are suitable for use in fluorescence microscopy, confocal laser scanning microscopy, fluorometry flow and cytometry, and can be employed as a nuclear counterstain. LIVE/DEAD staining cannot be performed for the direct staining of biofilms on surfaces because of interference between the stain and polysaccharides of the biofilm matrix and slime [177].

This method’s main downside involves having to observe a statistically relevant portion of the sample, which is representative of the whole population. Overall, the method provides only semiquantitative results because the total count of bacterial cells is not possible [178]. Nevertheless, this inconvenience can be prevented by employing imaging software, such as cellSens^®^, which can count and measure cells depending on the staining cell. 

#### 5.2.4. Different Fluorescents Stainings

The application of fluorescent stains to cells and food soil can be useful for the quantitative analysis of surface cleanability. Thus, the stain combination and working concentration are essential for assessing the hygienic conditions of surfaces [179] or testing disinfectant efficacy against bacteria. Different methods can be followed to visualise and differentiate cells and organic matter. The staining techniques to measure surface coverage by the two stains by image analysis are highlighted [180] using DAPI and Rhodamine B, DAPI and Fluorescein, or non-specific stains, such as acridine orange, and are also available and specific for particular organic matter [180], and/or for microorganisms [181]. 

The use of different staining types can be explained by the results obtained in each study after checking the best biofilm and cells staining. These results depend on bacterial species, residual organic matter on surfaces, pH, disinfectant, etc. Whitehead et al. [182] conducted a large study with different dyeing, and concluded that the best combination was DAPI (CAS no. 28718-90-3, λ_ex_ 340 nm, λ_em_ 488 nm, blue) and Rhodamine B (CAS no. 81-88-9, λ_ex_ 553 nm, λ_em_ 627 nm, red), as it allowed the quantitative determination of *L. monocytogenes* and whey on a surface with fluorescent staining under epifluorescence microscopy. It is also useful for demonstrating the hygienic status of surfaces (Figure 7 and Figure 8). The other tested staining procedures were unsatisfactory, or only slightly so, for distinguishing between viable and dead cells [182]. 

**Figure 6:**
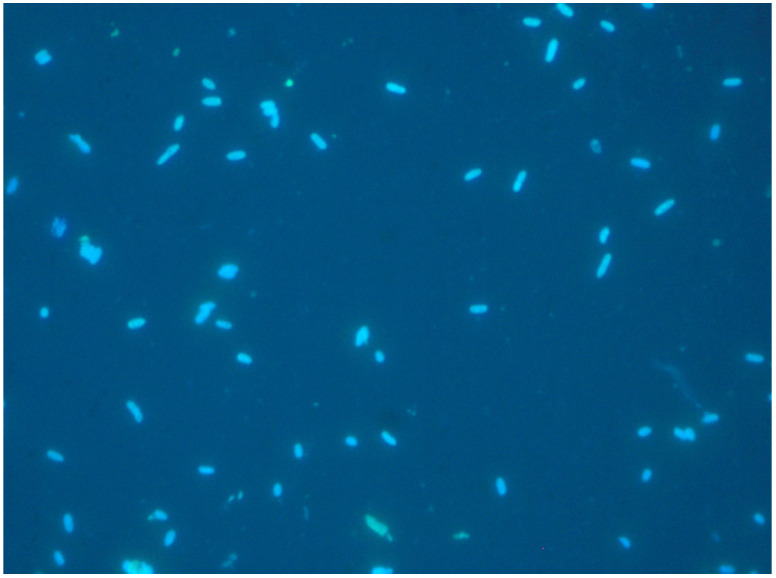
*Pseudomonas aeruginosa* biofilm on the stainless steel coupons. Stained with DAPI (0.1 mg/mL; 10 μL) (Magnification ×100).

DAPI staining is suitable for studying cell viability in planktonic situations (initial attachment) and biofilms attached to surfaces (proliferation and growth–maturation) (Figure 6). Nevertheless, coculture biofilm studies need to spatially discriminate between species, and classic methods, such as crystal violet (CV), SYTO9/propidium iodide, and DAPI staining are insufficient given their non-specific nature [183], and selectively bind to each species. This burden can be overcome by applications, such as mutants expressing green fluorescent protein (GFP) [182,184], fluorescently labelled antibodies [185], and fluorescence in situ hybridisation (FISH). 

**Figure 7:**
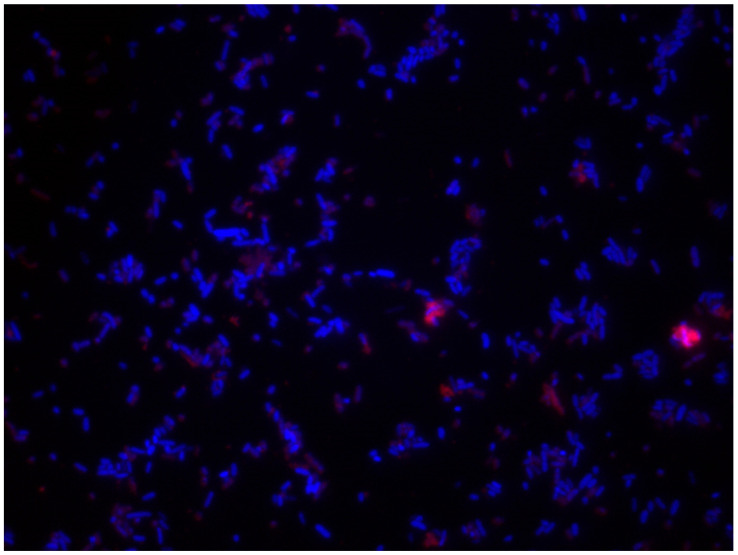
*Pseudomonas fluorescens* biofilm (24 h) on the stainless steel. Coupons stained with DAPI (0.1 mg/mL; 10 μL) and Rhodamine B (0.1 mg/mL; 10 μL) (Magnification ×100).

**Figure 8:**
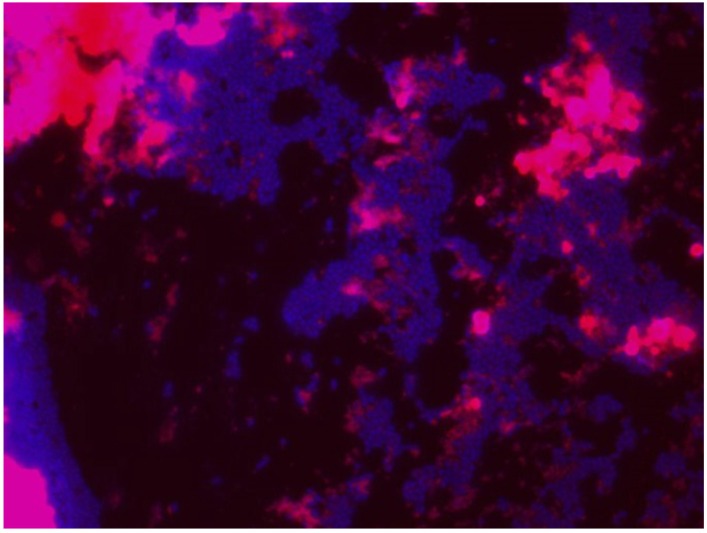
*Pseudomonas fluorescens* biofilm (7 days) on the stainless steel. Coupons stained with DAPI (0.1 mg/mL; 10 μL) and Rhodamine B (0.1 mg/mL; 10 μL) (Magnification ×100).

The DAPI/Rhodamine B combination in biofilms offers the best resolution and quantification power between cells and organic matter (Figure 6, Figure 7 and Figure 8). Several authors, such as Almeida et al. [183], have applied peptide nucleic acid fluorescence in situ hybridization (PNA FISH) combined with DAPI as a steady method to evaluate, validate, quantify, and characterise the initial adhesion and biofilm formation of three microorganisms: *Salmonella enterica*, *Listeria monocytogenes* and *Escherichia coli.*

#### 5.2.5. Confocal Laser Scanning Microscopy (CLSM)

Confocal laser scanning microscopy (CLSM) is an optical microscope equipped with a laser beam that is particularly useful for examining thick samples like microbial biofilms. Samples are stained with specific fluorescent dye insofar as the fluorescent light from the illuminated spot is collected on the objective and transformed by a photodiode into an electrical signal to be computer-processed [160] given the complexity of the microbial biofilm’s extracellular matrix formed by heterogeneous compounds: polysaccharide, lipids, enzymes, extracellular DNA, and proteins [186]. 

However, no fluorescence labelling method is currently available for visualising the whole biofilm matrix owing to its different compositions, which depend on each bacterium and environmental condition, which means that each matrix component must be individually stained. Unfortunately, however, a general stain for polysaccharides does not exist because the chemical structure of matrix polysaccharides differs between distinct bacteria: Gram + and Gram− [186].

Extracellular DNA has been related to bacterial attachment and early biofilm formation stages in many species across the phylogenetic tree. These findings were discovered by employing combined stains, such as PicoGreen^®^ and SYTOX^®^, PI, 1,3-dichloro-7-hydroxy-9,9-dimethyl-2(9H)-acridinone (DDAO), TOTO^®^-1, TO-PRO^®^ 3. Most reports employed DDAO for staining eDNA in biofilms after the first publications by Allesen-Holm et al. [187] and Conover et al. [188]. Excellent efficacy has been reported for TOTO^®^-1, SYTOX^®^ Green, while PI provides the most reliable results. TO-PRO^®^-3 and DDAO are not completely cell-impermeant [189].

With biofilm proteins, which may sometimes be more important than polysaccharides, this occurs in cell wall-anchored proteins in *Staphylococcus aureus* and *S. epidermidis,* and contributes to aggregation by homophilic interactions [190], or interacts with matrix components that originate from the host, such as fibronectin, collagen, or fibrin [191]. These biofilm proteins can be visualised with strains FilmTracer™ SyPro^®^ [192]. Several proteins also play a key role in the *P. aeruginosa* biofilm matrix, such as CdrA and others, perform functions that range from nutrient acquisition to protection from oxidative stress [193]. Moreover, serine-protease inhibitor ecotin has been identified as a matrix protein that binds to Psl [194].

Nowadays, confocal microscopy is a relevant tool for studying the structure of biofilms thanks to its excellent real-time visualisation capability of fully hydrated living samples. The limitation of light microscopy’s spatial resolution is improved by a fluorescence technique and by coupling CLSM with other imaging techniques [157]. The PNA FISH and CLSM combination allows the spatial organisation of and changes in specific members of complex microbial populations to be studied without disturbing the biofilm structure [195,196].

### 5.3. Raman Microscopy (RM)

This non-destructive analytical technique provides fingerprint spectra with the spatial resolution of an optical microscope [197]. This original technique permits the quantitative, label-free, non-invasive, and rapid monitoring of biochemical changes in complex biofilm matrices with high sensitivity and specificity [198]. Raman spectra studies are characterised by high specificity, and by usually revealing sharper clearer bands than IR spectra, and a small water background. Compared to IR microscopy, excitation with visible light can be employed in Raman spectroscopy, which allows standard optics to be utilised. Other advantages include its application to characterise and identify different biological systems (fungi, bacteria, yeasts) because all biologically associated molecules (e.g., nucleic acids, proteins, lipids, carbohydrates) exhibit distinct spectral features [197]. Therefore, Ivleva et al. [197] analysed seven different specific microorganisms by RM to characterise microorganisms in biofilms. 

Another author evaluated the antibiotic effect on biofilms [198], and the oxidation of graphene as antibacterial activity against the *Pseudomonas putida* biofilm with variable ages [199].

### 5.4. Scanning Electron Microscopy (SEM)

Scanning electron microscopy (SEM) provides useful information about size, shape, and localisation in the biofilms of single bacteria, and in biofilm formation process steps about bacterial interactions and EPS production [200]. Surface topography has been widely discussed as a parameter that influences microbial adhesion. In line with this, the experiments by Kouider et al. [201], which employed SEM to establish the effect of stainless steel surface roughness on *Staphylococcus aureus* adhesion, revealed that the adhesion level largely depends on substrate roughness with a maximum at Ra = 0.025 µm and a minimum at Ra = 0.8 µm. [202]. Mallouki et al. studied the anti-adhesive effect of fucans by SEM and a MATLAB programme to determine the number and characteristics of adhered cells [203].

SEM has been extensively used to qualitatively observe biofilm disruption owing to its high resolution, and is usually applied in combination with biological assays of biofilm removal efficiency [204,205]. With SEM images, simple thresholding cannot often be implemented because biofilm normal surface intensity values are similar due to the same effective contrast seen by SEM. Rough (textured) biomaterial surfaces complicate image analyses, and advanced segmentation methods, such as semi-supervised machine-learning techniques, are usually needed [206]. The biofilm might be segmented from the surface using the Trainable Weka Segmentation plugin, which utilises a collection of machine-learning algorithms for segmentation purposes [207]. 

**Figure 9:**
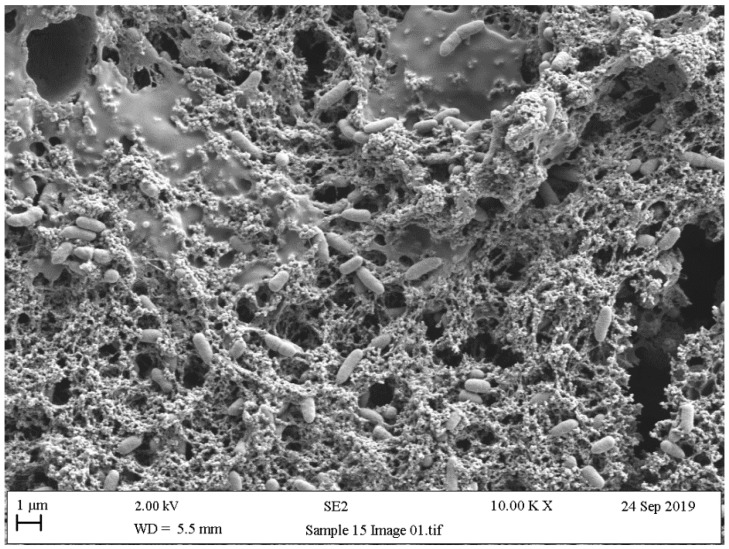
Scanning electronic microscopy (SEM) images of stainless steel of 3-day biofilms formed by *Pseudomonas fluorescens*.

**Figure 10:**
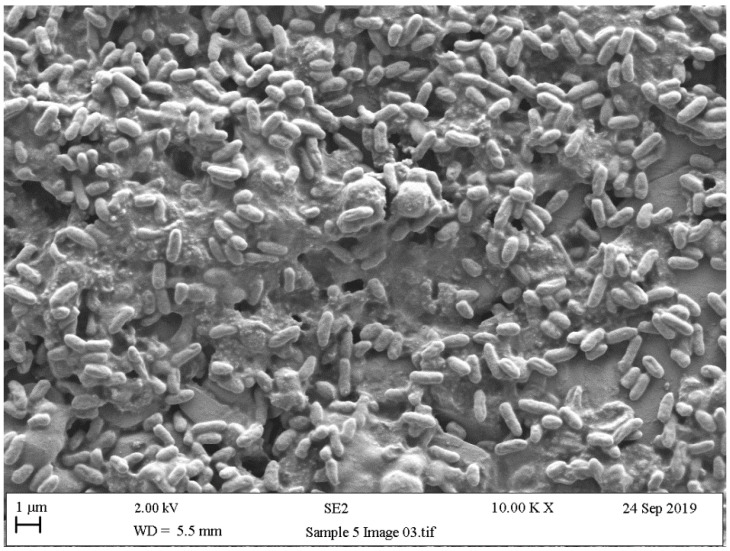
Scanning electronic microscopy (SEM) images of stainless steel of 7-day biofilms formed by *Pseudomonas fluorescens.*

**Figure 11:**
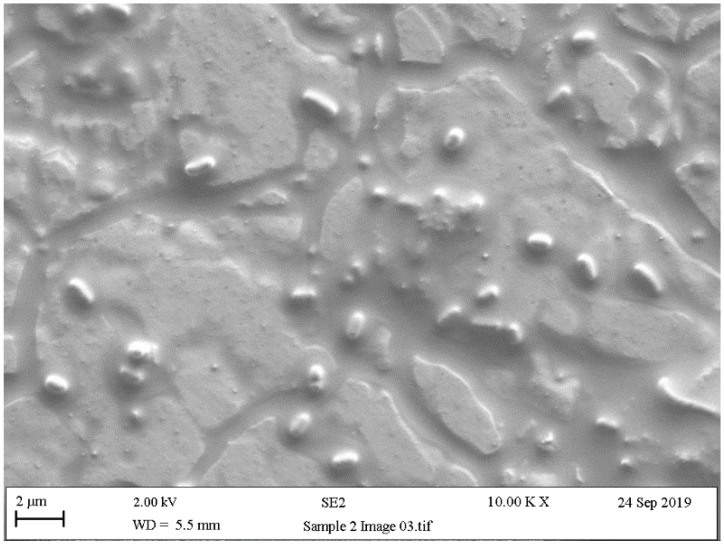
Scanning electronic microscopy (SEM) images of stainless steel of 7-day biofilms formed by *Pseudomonas fluorescens* after treatment with peracetic acid (250 ppm) for 15 min.

As with other previously mentioned techniques, SEM is a widely used resource for confirming the presence of bacteria and the exopolysaccharide matrix when studying biofilms (Figure 9, Figure 10 and Figure 11). These studies usually obtain SEM results and are supplemented with the results of other techniques like confocal [208,209], surface-enhanced Raman scattering (SERS) spectroscopy [210], epifluorescence microscopy (DAPI/Rhodamine B), and contact plates [211].

### 5.5. Microbiological Methods

The estimation of the total number of organisms (total viable count) is the most widely used technique to estimate biofilm viable cells. This count is done on agar media and its result is colony-forming units (CFU). Based on the serial dilution series approach followed to quantify microorganisms, this technique is easy and requires no special equipment [158]. Surface samples (stainless steel, plastic, rubber coupons) with biofilms are analysed by swab or sonication, and transferred to agar plates. This culture medium can be specific for either the studied species or non-specific species (plate count agar media).

Several authors like [212] discovered that some bacterial species can enter a distinct state called the viable, but non-culturable (VBNC) state. These living cells have lost the ability to grow on plate agar media. However, this method has serious drawbacks and limitations [213]: (i) the fraction of detached live cells may not be representative of the initial biofilm population; (ii) a subpopulation of biofilm cells can be viable, but non-culturable (VBNC), and cannot be detected by the CFU approach for the CFU estimation of the recovery and quantification of viable biofilm cells. Several authors, such as Cerca et al. [214] and Olivera et al. [215], have proposed applying flow cytometry coupled with a few possible fluorophores as an alternative to the total viable count from biofilms because flow cytometry solves both CFU counting limitations by distinguishing total, dead, and VBNC.

The total viable count technique is fundamental for the evolution of biofilm studies, as are studies about the efficacy of industrial disinfectants and increased resistance to the application of different disinfectants. Table 2 shows some results of disinfectant efficacy against several bacterial species.

## 6. Conclusions

Biofilms have become a major environmental microbiology concern in the food industry over the last 30 years. This topic is prominent due to the potential for contamination of food from biofilms; they are responsible for more than 20% of food poisoning cases and for being up to 1000-fold more tolerant to antibiotics than their planktonic counterparts [219].

Many bacterial species have the ability to form biofilms, such as microbial subsistences (when faced with hostilities from the environment), antibiotics, and disinfectants. For these reasons, cleaning and disinfecting in the food industry must bring about changes that favour eliminating biofilms, because once they form, the resulting costs and risks will be very high. As previously discovered in many publications, the ability of bacteria to form biofilms is greater than the discoveries. Thus, they must be eliminated. The advancement of new, non-destructive technologies (e.g., laser dissection) to study biofilms and their results should be applied to biofilm diagnoses in the food industry, to better understand the physiological anatomy of microbes and biofilms, and future applications in the food industry.

## Figures and Tables

**Table 1:** Biofilm-forming pathogens in the food industry.

Pathogen	Characteristics	Contaminated Food	Examples of Harmful Spoilage Effects	References
*Bacillus cereus*	Gram-positive, spore-forming, anaerobic, facultative anaerobic	dairy products, rice, vegetables, meat	diarrhoea and vomiting symptoms	[39,40]
*Campylobacter jejuni*	Gram-negative, aerobic and anaerobic	animals, poultry, unpasteurised milk	bloody diarrhoea, fever, stomach cramp, nausea and vomiting	[41,42,43]
*Escherichia coli*	Gram-negative, rod-shaped	raw milk, fresh meat, fruits and vegetables	diarrhoea outbreaks and haemolytic uremic syndrome	[44]
*Listeria monocytogenes*	Gram-positive, rod-shaped, facultative anaerobic	dairy products, meat, ready-to-eat products, fruit, soft cheeses, ice cream, unpasteurised milk, candied apples, frozen vegetables, poultry	listeriosis in the elderly, pregnant women and immune-compromised patients	[45,46]
*Salmonella Enterica*	Gram-negative, rod-shaped, flagellate, facultative aerobic	Poultry meat, bovine, ovine, porcine, fish	can cause gastroenteritis or septicaemia	[47,48]
*Staphylococcus aureus*	Gram-positive, non-spore forming, non-motile, facultative anaerobic	meat products, poultry, egg products, dairy products, salads, bakery products, especially cream-filled pastries and cakes, and sandwich fillings	methicillin resistance, can cause vomiting and diarrhoea	[49,50]
*Pseudomonas* spp.	psychrotrophic, motile, Gram-negative rod-shaped	fruits, vegetables, meat surfaces and low-acid dairy products	produces blue discolouration on fresh cheese.	[17]
*Geobacillus stearothermophilus*	thermophilic, Gram-positive, spore-forming, aerobic or facultative anaerobic	dried dairy products	production of acids or enzymes leading to off-flavours	[51,52]
*Anoxybacillus flavithermus*	thermophilic organism, Gram-positive, spore-forming, facultatively anaerobic, non-pathogenic	dried milk powder	an indicator of poor hygiene	[53,54]
*Pectinatus* spp.	Gram-negative, non-spore-forming, anaerobic	beer and brewery environment	rapid cell growth makes beer turbid and smells like rotten eggs due to production of sulphur compounds	[23]

**Table 2:** Resistance of several bacterial species to disinfectant on different material surfaces.

Bacterial Species	Disinfectant	ppm or %	Surfaces	Biofilm. Log Reduction CFU	Reference
*S. aureus*	Sodium hypochlorite	250	Stainless steel/PP	4.5/4.4	[212]
*Cronobacter Sakazakii*	Sodium hypochlorite	250	Stainless steel/PP	3.7/3.9	[216]
*S. Typhimurium*	Sodium hypochlorite	250	Stainless steel/PP	5.82/6.1	[216]
*S. aeruginosa*	Sodium hypochlorite	250, 500	Stainless steel 316	2/100%	[217]
*S. aeruginosa*	Sodium hypochlorite	750, 1000	Stainless steel 316	100%/100%	[217]
*B. cereus*	NaOH and HNO_3_, 65 °C	1%	CIP dairy	2	[218]
*Enterococcus faecium*	Sodium hypochlorite	100	Stainless steel	3	[98]
*E. faecium*	Peracetic acid	300	Stainless steel	4	[98]
*Rhodococcus erythropolis*	Alkyl amine	1–1.3%	Stainless steel	>5	[103]
*R. erythropolis*	Peracetic acid	0.2%	Stainless steel	0.48	[103]
*R. erythropolis*	Sodium hypochlorite	0.5–1%	Stainless steel	4.51	[103]
*R. erythropolis*	QAC	200	Stainless steel	>5	[103]
*Sphingomonas* sp.	Alkyl amine	1–1.3%	Stainless steel	>5	[103]
*Sphingomonas* sp.	Peracetic acid	0.2%	Stainless steel	>5	[103]
*Sphingomonas* sp.	Sodium hypochlorite	0.5–1%	Stainless steel	>5	[103]
*Sphingomonas* sp.	QAC	200	Stainless steel	>5	[103]
*Methylobacterium rhodesianum*	Alkyl amine	1–1.3%	Stainless steel	4.48	[103]
*M. rhodesianum*	Peracetic acid	0.2%	Stainless steel	>5	[103]
*M. rhodesianum*	Sodium hypochlorite	0.5–1%	Stainless steel	0.01	[103]
*M. rhodesianum*	QAC	200	Stainless steel	0.64	[103]
*L. monocytogenes*	Sodium hydroxide	0.5%	Rubber	0.66	[112]
*L. monocytogenes*	QAC	0.5%	Rubber	1.72	[112]
*L. monocytogenes*	Sodium hypochlorite	0.5%	Rubber	1.79	[112]
*L. monocytogenes*	Peracetic acid	0.5%	Rubber	5.10	[112]
*L. monocytogenes*	Sodium hydroxide	0.5%	Polypropylene	1.20	[112]
*L. monocytogenes*	QAC	0.5%	Polypropylene	2.57	[112]
*L. monocytogenes*	Sodium hypochlorite	0.5%	Polypropylene	2.74	[112]
*L. monocytogenes*	Peracetic acid	0.5%	Polypropylene	6.62	[112]
*L. monocytogenes*	Sodium hydroxide	0.5%	Stainless steel	1	[112]
*L. monocytogenes*	QAC	0.5%	Stainless steel	4.06	[112]
*L. monocytogenes*	Sodium hypochlorite	0.5%	Stainless steel	1.97	[112]
*L. monocytogenes*	Peracetic acid	0.5%	Stainless steel	6.63	[112]
*L. monocytogenes*	Sodium hydroxide	0.5%	Aluminium foil	0.52	[112]
*L. monocytogenes*	QAC	0.5%	Aluminium foil	5.1	[112]
*L. monocytogenes*	Sodium hypochlorite	0.5%	Aluminium foil	3.84	[112]
*L. monocytogenes*	Peracetic acid	0.5%	Aluminium foil	6.54	[112]
*L. monocytogenes*	Benzalkonium chloride	100–10,000	Polystyrene	1–7	[112]
*L. monocytogenes*	Benzalkonium chloride	10	Polystyrene	100%	[170]

Colony-forming units (CFU)**;** cleaning-in-place (CIP)**;** polypropylene (PP)**;** quaternary ammonium compounds (QAC).

## Data Availability

Not applicable.
